# Embryoid Body Test: A Simple and Reliable Alternative Developmental Toxicity Test

**DOI:** 10.3390/ijms252413566

**Published:** 2024-12-18

**Authors:** Inho Hwang, Eui-Bae Jeung

**Affiliations:** Laboratory of Veterinary Biochemistry and Molecular Biology, College of Veterinary Medicine, Chungbuk National University, Cheongju 28644, Republic of Korea; darkpower777@nate.com

**Keywords:** embryoid body test (EBT), developmental toxicity, alternative test

## Abstract

The increasing emphasis on animal welfare and ethics, as well as the considerable time and cost involved with animal testing, have prompted the replacement of many aspects of animal testing with alternative methods. In the area of developmental toxicity, the embryonic stem cell test (EST) has played a significant role. The EST evaluates toxicity using mouse embryonic stem cells and somatic cells and observes the changes in heartbeat after cardiac differentiation. Nevertheless, the EST is a relatively complex testing process, and an in vitro test requires a long duration. Several attempts have been made to develop a more straightforward testing method than the EST, with improved reproducibility and accuracy, leading to the development of the embryoid body test (EBT)**.** Unlike the EST, which involves cardiac differentiation stages, the EBT verifies toxicity by measuring the changes in the area of the embryoid body. Despite its short testing period and simple procedure, the EBT offers high accuracy and reproducibility and is fully validated through two rounds of validation, making it ready for practical application. The EBT is expected to play a crucial role in the rapidly increasing demand for alternative methods to animal testing, particularly for screening early developmental toxicity.

## 1. Introduction

### 1.1. Characteristics of Developmental Toxicity

The embryonic developmental phase is high unstable due to rapid changes, making it vulnerable to toxic substances during implantation, differentiation, and fetal development [1]. Cell division progresses the embryo from two germ layers (ectoderm and endoderm) to three (ectoderm, mesoderm, and endoderm), initiating organ development (Table 1) [2]. Therefore, early developmental toxicity often impacts multiple essential organs, potentially causing severe or fatal outcomes for the embryo [3].

Owing to these considerations, pregnant women should be particularly cautious of exposure to substances with embryonic developmental toxicity during early pregnancy (Table 2). Avoiding exposure to alcohol, retinoids (e.g., isotretinoin), and heavy metals is crucial during early pregnancy [12,13,14]. Alcohol crosses the placenta, leading to fetal alcohol spectrum disorders (FASDs) and embryonic developmental deficiencies [12]. Retinoids used for acne can cause underdevelopment or fetal organ malformations [13]. Exposure to heavy metals like mercury can impair the development of organs in the fetus, such as the brain, kidneys, and liver [14]. In addition, plastics may release endocrine-disrupting chemicals (EDCs), like phthalates, causing developmental toxicities via oxidative stress and epigenetic changes [15].

Certain medications can induce even more substantial fetal developmental toxicity, including valproic acid as an anticonvulsant, lithium for bipolar disorder, angiotensin-converting enzyme (ACE) inhibitors for hypertension, non-steroidal anti-inflammatory drugs (NSAIDs), methotrexate for autoimmune diseases, the anticoagulant warfarin, and thalidomide, which was once used to treat morning sickness but is now used as an anti-cancer agent [16,17,18,19,20,21,22]. Angiotensin II plays a vital role in the development of fetal kidneys and heart; therefore, ACE inhibitors can lead to impaired renal formation and function in the fetus, resulting in renal failure, cardiovascular malformations, and fetal hypotension. This, in turn, can cause pulmonary development disorders as well as inhibited skull and brain development in the fetus [16]. Lithium inhibits the intracellular signaling pathways, particularly the glycogen synthase kinase-3 (GSK-3) pathway, affecting the migration and differentiation of neural crest cells during the early stages of heart development, which can lead to cardiac malformations; for example, Ebstein’s anomaly is a congenital heart defect in which the tricuspid valve does not form properly, resulting in abnormal blood flow, and it is strongly associated with lithium use [17]. Valproic acid inhibits the folic acid metabolism, increases oxidative stress-induced cellular damage, and acts as an histone deacetylase (HDAC) inhibitor, causing epigenetic changes that can lead to neural tube defects, heart malformations, and cleft lip/palate [18]. NSAIDs, by inhibiting cyclooxygenase (COX), can cause various abnormalities in the fetus’s condition and development [19]. Methotrexate, an antifolate, primarily disrupts cell division and DNA synthesis, leading to skeletal malformations, heart defects, and neurodevelopmental malformations; in addition, it can result in low birth weight or growth delays because of impaired placental function [20]. Warfarin inhibits vitamin K, impacting all the related developmental processes, which can lead to respiratory distress, skeletal malformations, neurological defects, multiple organ malformations, and neonatal death [21]. Thalidomide and related drugs cause deficiencies in the development of the nervous system, heart, and limbs [22].

Infectious diseases also present risks; for example, cytomegalovirus (CMV) infection can lead to nervous system developmental disorders, as well as impairments such as vision and hearing loss [23]. Infection with the rubella virus causes congenital rubella syndrome (CRS), which can result in severe congenital multiple systemic disabilities [24].

**Table 2:** Several known representative developmental toxic substances and their major actions.

Teratogenic Agent	Features/Primary Use	Developmental Toxicity
Alcohol	Alcohol consumption	Fetal alcohol spectrum disorders (FASDs) including birth defects, craniofacial anomalies, central nervous system (CNS) dysfunction, and growth retardation [12].
Retinoic acid (vitamin A derivatives)	Acne treatment, leukemia treatment	Fetal retinoid syndrome (FRS) including birth defects, craniofacial malformation, CNS abnormalities, heart defects, and thymus gland abnormalities [13].
Heavy metals (e.g., mercury and lead)	Industrial use, fish consumption	Causes neurotoxicity, affecting brain development and kidney/liver function [14].
Phthalates	Plasticizer	Increased risk of low birth weight, preterm birth, and mental disorders (autism and ADHD) [25,26].
ACE inhibitors (e.g., enalapril)	Hypertension, heart failure treatment	Renal failure, lung dysplasia, oligohydramnios (low amniotic fluid) cranial hypoplasia, limb contractures, and prolonged hypotension [16].
Lithium	Mental illness treatment	Ebstein’s anomaly (heart defect affecting the tricuspid valve) and overall cardiac defects [17].
Anti-epileptic drugs (e.g., valproic Acid)	Seizure, bipolar disorder treatment	Neural tube defects (e.g., spina bifida), heart defects, cleft lip/palate, and developmental delays [18].
NSAIDs (e.g., ibuprofen)	Anti-inflammatory treatment	Cardiac septal defect, gastroschisis, colostomy and diaphragmatic hernia, miscarriage, growth retardation, ductus arteriosus constriction, reduced renal perfusion, oligohydramnios, and increase in bleeding tendencies [19].
Methotrexate	Cancer, autoimmune disease (e.g., psoriasis) treatment	Neural tube defects, skeletal abnormalities, growth retardation, and spontaneous abortion [20].
Warfarin (e.g., coumadin)	Anti-thrombotic treatment	Respiratory distress, skeletal abnormalities (e.g., nasal hypoplasia, stippled epiphyses), calcification of hyoid and thyroid cartilages, neurological defects, and neonatal death [21].
Thalidomide	Anti-cancer treatment (multiple myeloma)	Absence of the limb (amelia) or proximal limb elements (phocomelia), heart, ears, eyes, kidney, and genital defects [22].
Cytomegalovirus	Viral infection	Microcephaly, periventricular calcification, cerebellar hypoplasia, eye abnormalities, hearing loss, seizure, and epilepsy [23].
Rubella virus	Viral infection	Congenital rubella syndrome (CRS), including multiple systemic complications such as heart defects, ocular abnormalities, hearing defects, microcephaly, hepatitis, bone lesions, pneumonitis, and thyroid disorders [24].

The thalidomide incident is a representative example of embryonic developmental toxicity. Although thalidomide is currently used as a treatment for multiple myeloma through ongoing research, it was originally developed and used in the 1950s to treat morning sickness in pregnant women; thalidomide exerts toxic effects such as inhibiting angiogenesis, inducing oxidative stress, and suppressing Sall4 gene expression, affecting the development of major organs and limbs [27]. These toxic effects led to miscarriages and stillbirths when major organ abnormalities occurred. Even in surviving fetuses, thalidomide caused phocomelia, a condition characterized by severely underdeveloped limbs. Survivors affected by thalidomide in the womb lived with limb development deficiencies throughout their lives [28]. Hence, the fetal developmental period is critical for the formation of organs, limbs, and other body structures. The toxic effects experienced during this period cannot be overcome through growth after birth, requiring substantial caution.

### 1.2. Animal Test in Embryonic Developmental Toxicity Test

Appropriate animal testing must be considered to prevent harm from developmental toxicity, such as in the thalidomide case. An in vitro test cannot fully capture all biological side effects. Therefore, animal testing that considers the genetic sequences, development, and morphological characteristics of different species is essential. Regarding developmental toxicity, the organization for economic cooperation and development (OECD) recommends testing methods that can confirm embryonic developmental toxicity, such as toxicity guideline (TG) 414 (Prenatal Developmental Toxicity Study), 415 (One-Generation Reproduction Toxicity), 416 (Two-Generation Reproduction Toxicity), 421 (Reproduction/Developmental Toxicity Screening Test), 422 (Combined Repeated Dose Toxicity Study with the Reproduction/Developmental Toxicity Screening Test), 426 (Developmental Neurotoxicity Study), and 443 (Extended One-Generation Reproductive Toxicity Study) (Table 3) [29,30,31,32,33,34,35].

Currently, the types of developmental tests conducted for new drug approval mainly include embryo–fetal development tests, multi-generation reproductive toxicity tests, and pre- and postnatal developmental toxicity tests [36]. Although the methods used for these tests differ, they commonly use animals, such as rodents and rabbits, to evaluate the reproductive function, pregnancy, birth, and teratogenicity. Despite the advantage of verifying biological reactions through cell tests and genetic research that cannot be understood entirely without animal testing, the process is time-consuming and expensive [37]. Moreover, the growing emphasis on animal ethics is driving the trend toward minimizing animal experiments, which presents a challenge. One perspective is that research using systems that are as similar to humans as possible is essential to minimize harm to humans. This contrasts with the view that all side effects cannot be known through animal testing because of species differences and living conditions, deeming animal sacrifices unnecessary [38,39,40]. As a result, the direction is gradually moving toward reducing animal testing, and alternative methods to animal testing are being developed.

### 1.3. Alternative Animal Tests

The need for alternative animal testing methods to reduce animal experiments is growing. According to the 2023 Non-Animal Alternative Testing Market Report, the global market size for alternative animal testing methods is estimated to be at USD 1.7 billion, and it is projected to grow to USD 4.5 billion by 2032 with a CAGR of 10.3% [41]. In particular, rapid changes occur in a short time during the embryonic developmental period, and the related impacts can be fatal or irreversible, as mentioned previously [42]. Thus, toxicity evaluation is crucial, and various alternative testing methods are being developed or used (Table 4). In vitro studies are based primarily on cytotoxicity tests using methods such as MTT or CCK assays on cultured cells [43]. They also assess developmental toxicity by checking for embryoid body formation or differentiation.

Three-dimensional assays, such as spheroid or organoid formation, are used to simulate environments that resemble actual biological conditions more closely [44,45]. In silico assays use computer simulations, including the following: quantitative structure–activity relationship (QSAR) modeling, which analyzes the structural characteristics and toxicity relationships of chemicals to predict developmental toxicity; read-across, which uses toxicity information from similarly structured chemicals to predict the toxicity of new substances; and toxicokinetic models, which simulate the absorption, distribution, metabolism, and excretion processes of chemicals in the body to predict developmental toxicity [46,47]. In addition, in chemico tests using chemical reactivity and protein binding assays, multi-omics approaches through genomics, transcriptomics, proteomics, metabolomics, imaging, or non-invasive techniques are used as alternative animal testing methods [48,49]. MPSs (Microphysiological Systems) are an innovative technology that combine 3D culture techniques with microfluidic technology to recreate the interactions and microenvironment of human tissues or organs outside the body [50]. Ex vivo studies, while using animal-derived tissues and potentially conflicting with the goal of replacing animal testing, can reduce animal sacrifice significantly by allowing for multiple tests from a single animal while also benefiting from the use of real animal tissues [51,52,53]. The use of zebrafish, a vertebrate that allows for more affordable and efficient testing on large numbers of specimens, as well as invertebrates like C. elegans and drosophila, for various experiments, has increased [54,55,56,57,58,59].

**Table 4:** Various alternative animal testing methods and their characteristics.

Method.	Description	Advantages	Limitations	Applications
In vitro (2D) [43]	Experiments conducted using two-dimensional (2D) cell cultures.	Relatively cost-/time-efficient and simple.	Artificial cultural conditions, impairs intracellular signaling, lack of cell interaction, homeostasis, and total organism integration.	Screening studies (e.g., MTT cytotoxicity assay) and mechanistic investigations.
In vitro (3D) [45]	Using three-dimensional (3D) culture system.	More cost-/time-efficient than animal test; recapitulation of developmental biology and human physiology.	More labor-intensive and time-consuming than 2D test, lack of vascularization, low reproducibility and standardization.	Organoids and spheroid toxicity assays.
In silico [46,47]	Predictive tests conducted by computer, such as modeling or simulation.	Highly cost- and time-efficient, predictive capability, integration with other techniques, and leveraging existing knowledge.	Validation and accuracy, complexity of biological system, regulatory acceptance, data availability and quality, and interdisciplinary expertise.	Toxicity prediction (e.g., QSAR models), molecular docking, and drug design.
In chemico [48,49]	The use of non-biological chemical reactivity methods as replacements for in vivo assays.	Mechanistic insights into toxicity, standardization, reproducibility, cost-/time-efficient, and applicability across various toxicity endpoints.	Limited reflection of in vivo environments, false outcomes, restricted applicability domain, and complementary testing needs.	Chemical safety assessment and skin sensitization tests (e.g., Direct Peptide Reactivity Assay (DPRA)).
Microphysiological Systems (MPSs) [50]	A multicellular system designed to simulate the physiological environment of human tissues and organs.	Closely mimics human biology, providing high predictive accuracy, customization, and flexibility.	Technically complex: requires specialized equipment and expertise (high cost).	Organoids and organs-on-a-chip.
Ex vivo [51,52,53]	Tissues or organs from living organisms are extracted, maintained under controlled conditions, and tested.	Minimum alteration and variation, ethically restricted test can be conducted, comparable results with reduced animal use.	Not fully recapitulate physiological conditions, and the tissue extraction process involves the sacrifice of animals.	Ex vivo lung perfusion, rat aortic ring assay, patient-derived cancer models, and tissue response studies.
Zebrafish Model [54,55]	Use of zebrafish embryos, larvae, and adults as alternative animal models depending on the purpose.	Easy and cost-efficient handling, fast development, small size, transparency, genetic similarity, and potential to perform high-throughput assays.	Physiological differences between fish and humans, lack of certain organs, environmental sensitivity, and regulatory acceptance.	Developmental biology, toxicity test (cardio, neuro, hepato, and nephron), and xenograft.
Invertebrate [56,57,58,59]	Tests using invertebrate organisms, such as worms or insects.	Ethical concerns are reduced, cost-/time-efficient, diverse species, and ecological relevance.	Extrapolation challenge, limited knowledge, methodological limitations, and regulatory acceptance.	Caenorhabditis elegans toxicity model and drosophila brain disease model.

### 1.4. Embryonic Stem Cell Test

Although more appropriate testing methods exist depending on the objective, the most widely used methods are those that produce similar results to animal tests, are time- and cost-effective, and are methodologically accessible. Developed in the late 1990s by Professor Spielmann’s research team, the embryonic stem cell test (EST) is one of the most used alternative animal tests for developmental toxicity [60]. As the name suggests, it uses embryonic stem cells to assess the effects on cell differentiation and development. The test involves functional analysis through the cell viability of the formed embryoid bodies, differentiation markers for cardiac cells, and the observation of cardiac cell contraction activities. Although the testing period exceeds ten days, making it relatively long for an in vitro test, and the procedures for culturing stem cells and forming and differentiating embryoid bodies are quite complex, it saves time and expense compared to animal testing. Moreover, the EST shows relatively high relevance and is widely used for toxicity evaluation and chemical regulation tests [61].

The institution that developed the EST, ZEBET (the Center for Documentation and Evaluation of Alternative Methods to Animal Experiments), has continuously worked on validating and improving this method [62,63,64]. By combining fluorescence-activated cell sorting (FACS), the testing period was shortened, the accuracy improved, and it underwent validation twice by the European Centre for the Validation of Alternative Methods (ECVAM) [65,66]. Based on these efforts, the EST plays a crucial role as an alternative animal test for embryonic developmental toxicity. In Japan, the Japan Center for the Validation of Alternative Methods (JaCVAM) developed an EST method that uses embryoid bodies with an inserted Hand1-Luc gene to analyze the luciferase signals during cardiac cell differentiation [67]. This improved method reduces the test duration from over ten days to approximately five to six days and simplifies the process compared to the original EST. Although the EST and improved methods based on it have been developed and used, several challenges, such as long testing periods, reduced reproducibility due to complex processes, and the need for expensive instruments, have not been fully resolved. As alternative testing methods aim to be more accessible, there was a need to develop a more straightforward method that could be used anywhere, allowing anyone to obtain consistent results quickly. This paper introduces the testing method developed from this perspective and aims to provide a comprehensive overview of its development and validation process, applications, strengths and limitations, and future prospects.

## 2. The EBT—Development and Optimization

The embryoid body (EB) is a cell aggregate that forms when stem cells, primarily embryonic stem cells, spontaneously organize into a three-dimensional structure, providing a similar environment to that of early embryonic development [68]. The EB contains all three germ layers and plays a crucial role in mimicking early embryonic tissue formation, with vast differentiation potential and activation of cell signaling pathways [69]. The EB also supports cell-to-cell signaling and structural support through the formation of the extracellular matrix (ECM), which is essential for early development [70]. The EB, which has the characteristics of early embryonic developmental processes, produces similar results to those of animal experiments without directly conducting animal testing [71]. Consequently, the EB is used widely in research related to early embryonic development [72].

Kang et al. developed a more straightforward and reliable method for the alternative animal testing of embryonic developmental toxicity called the embryoid body test (EBT) [73]. The EBT method involves using mouse embryonic stem cells (ES-E14TG2a) and mouse embryonic fibroblast cells (3T3-L1). After treatment with a substance, the test evaluates the reduction in embryoid body area and the cytotoxicity to each cell line, combining results from three subtests. The EBT, while using embryoid bodies from embryonic stem cells like existing tests, is more straightforward and achieves more than 80% accuracy with high reproducibility and reliability [73]. The test has undergone validation during development and two subsequent validation phases, and annual studies on the toxicity of specific substances using this method have been reported [74,75,76,77,78].

### 2.1. Method Development

**Figure 1:**
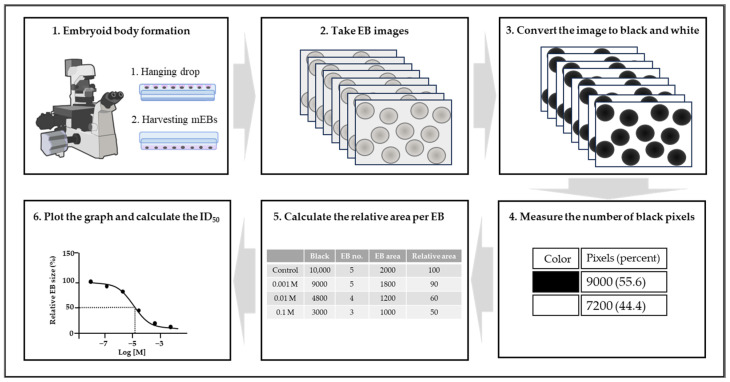
Method for measuring embryoid body (EB) size. 1. Collect EBs from hanging drops. 2. Capture 7~8 images of each group, ensuring that at least 5 EBs are included. 3. Convert the image to black and white. 4. Count the number of black pixels in the images. 5. Calculate the relative area per EB. 6. Plot the graph and determine the ID50.

The EBT is an improved method over the previously widely used EST as a developmental toxicity alternative to animal testing. The EST method involves evaluating cytotoxicity in mouse embryonic stem cells and somatic cells, as well as assessing the beating of differentiated cardiac cells, which was particularly time-consuming and complex [79]. The EBT method simplifies this by excluding the cardiac differentiation assessment, instead forming embryoid bodies and evaluating the changes in their area (Figure 1), resulting in a more straightforward and highly reproducible test [73].

The EBT uses mouse embryonic stem cells (ES-E14TG2a) and mouse somatic cells (3T3), along with embryoid bodies, to assess the cytotoxicity of a test substance on each cell type and the toxicity on the formed embryoid bodies, with its unique aspect being the assessment of toxicity based on the area of the embryoid bodies rather than through differentiation, despite its similarity to existing alternative tests for developmental toxicity (Table 5) [73].

Generally, the simpler the testing method, the higher the reproducibility, making it accessible to a broader range of users despite the challenge of establishing a correlation with actual toxicity, which was validated during the development phase using 21 well-known toxic substances by identifying biomarkers related to the embryoid body size, showing that toxicity suppresses the cell cycle and reduce the embryoid body area [73]. A comparative analysis with the existing EST revealed a strong correlation between post-differentiation beating and EBT embryoid body area, with the EBT predictive model (90.5% accuracy) outperforming the EST model (86.9% accuracy) in testing 21 substances [73]. While both the EST and EBT performed well, there were notable differences in the misclassification. The EST misclassified isoniazid, a non-toxic substance, as having moderate toxicity; lovastatin, a moderately toxic substance, as strongly toxic; and D-penicillamine, a strongly toxic substance, as moderately toxic [73]. On the other hand, the EBT shared the misclassification of D-penicillamine with the EST but correctly classified the other substances [73]. Among these substances, the in vivo classifications included six non-toxic substances such as sodium bicarbonate and ascorbic acid, weakly toxic substances like caffeine and aspirin, moderately toxic substances such as diphenhydramine and dexamethasone, and strongly toxic substances like methotrexate and retinoic acid [73]. Including cases where classifications differed across trials, the EBT demonstrated slightly higher accuracy, reinforcing its reliability and predictive strength.

### 2.2. Pre-Validation

A testing method must be validated during embryonic development. For a method to be accepted as an international standard for alternative developmental toxicity testing, it must yield consistent results regardless of the operator or the laboratory in which it is performed. In the embryonic development phase, the accuracy and mechanisms were validated using known toxic substances. In the first validation phase, 26 substances were tested to verify the intra-laboratory reproducibility, inter-laboratory transferability, and overall reproducibility by comparing the results from various researchers and laboratories [74].

Pre-validation was conducted under the direction of KoCVAM (Korean Centre for the Validation of Alternative Methods) and involved a validation management team (lead laboratory and participatory laboratories 1 and 2), a chemical management group, and a data analysis group. KoCVAM assigned code names to the test substances and provided them to the validation management teams. The biostatistician of the data analysis group analyzed the test results statistically based on the predictive model, verifying intra-laboratory reproducibility, inter-laboratory transferability, and inter-laboratory reproducibility, which were 84%, 92%, and 82%, respectively, proving the EBT to be an excellent and reliable alternative developmental toxicity test [74].

The testing method and result classification were improved during the pre-validation process, with the toxicity testing period for mouse stem cells and somatic cells set to 10 days, similar to the EST [74]. During validation, the testing period was reduced to four days, providing results equivalent to the 10-day test, while reducing errors from long-term culture and media changes, saving time and costs; additionally, the result classification was simplified from four stages (non, weak, moderate, and severe) to two stages (non-toxic and toxic) due to variations in toxicity depending on pregnancy stages (Table 6), significantly enhancing the accuracy and reproducibility of the test method [73,74].

### 2.3. Second-Phase Validation

In alternative animal testing methods, the accuracy of the predictive model is paramount; this accuracy improves as more data accumulate. The predictive model of the EBT method has been updated as new data from actual tests are added for increased accuracy, followed by a validation test (Table 7). The second-phase validation was conducted using the same team composition, leadership structure, and testing methods as the pre-validation test, with 35 substances, including 11 non-toxic and 24 toxic substances, used to evaluate the inter-laboratory reproducibility and predictive capability [75].

The toxicity is predicted using a formula that incorporates the IC_50_ values obtained from the cell viability tests of mESC and 3T3 cells and the ID_50_ values from the embryoid body toxicity test based on the area ratio [73]. The predictive model was updated by combining the results from 21 substances used in the development of the EBT and 108 data points from the pre-validation test, totaling 129 substance results. The accuracy of the predictive model built using this training set was confirmed to be 92%, with a sensitivity and specificity of 93% and 91%, respectively [75].

The reproducibility and predictive capability evaluations were conducted to verify the performance of the new predictive model. For the inter-laboratory reproducibility test, five common substances were provided to the lead laboratory and participatory laboratories 1 and 2. The lead laboratory and participatory laboratory 2 showed 80% accuracy, while participatory laboratory 1 showed 100% accuracy. The inter-laboratory reproducibility test results showed an accuracy, sensitivity, and specificity of 87%, 78%, and 100%, respectively [75]. The predictive capability of the model was assessed by giving 10 substances, consisting of 3 non-toxic and 7 toxic substances, to each of the three laboratories. The overall test results for the 30 substances showed an accuracy, sensitivity, and specificity of 80%, 81%, and 78%, respectively [75]. As a result, the updated predictive model, based on the toxicity classification results of 129 substances, was validated using 35 substances, confirming it to be a highly accurate testing method with more than 80% accuracy. Through two rounds of validation following its development, the EBT method achieved a level of completeness suitable for practical applications (Table 8).

## 3. Embryoid Body Test (EBT)—Significance and Utilization

### 3.1. Significance of the EBT

Completely replacing animal testing with alternative methods is challenging. Simulating the intricate biological interactions in a living system with a single artificial system is virtually impossible. Furthermore, although alternative methods may be simpler than animal testing, it is impractical to use all the available alternatives. Therefore, rather than aiming to replace animal testing completely, it is more reasonable to use appropriate alternative methods as a preliminary screening to prevent unnecessary animal testing and save time and expense. The most desirable tests are those that are quick, efficient, and reliably accurate.

**Figure 2:**
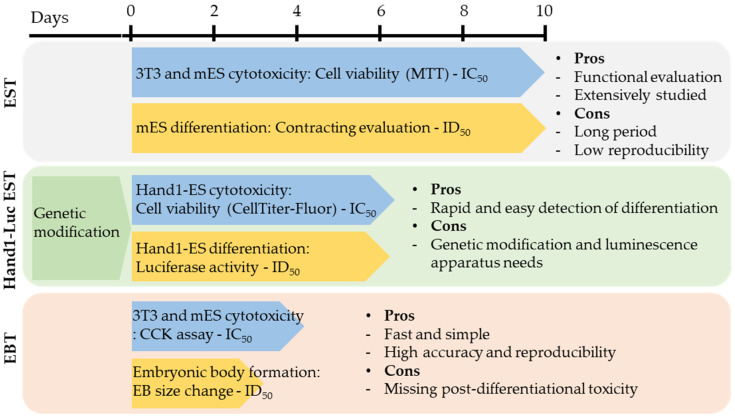
Experimental setup and timeline of the EST, Hand1-Luc EST, and EBT. The EST requires a 10-day testing period, making it relatively time-consuming and less reproducible, but it is the most extensively studied method. The Hand1-Luc EST offers the advantage of simplifying the detection of differentiation toxicity using luciferase; however, it requires luciferase measurement equipment, genetic modification for Hand1-Luc introduction, and maintenance of the cell line, which are notable drawbacks. The EBT, on the other hand, is the fastest and simplest method, offering higher reproducibility and accuracy. Nevertheless, it has the limitation of not providing direct post-differentiation results (e.g., beating activity).

Developmental toxicity testing involves significant animal sacrifice because of the uncertainty of obtaining sufficient subjects from conception to birth. The rapid and significant changes during this period necessitate a large number of subjects to ensure reliable results, leading to increased cost and time. In particular, in the drug screening stage, where development directions are selected from many substances, the pre-application of alternative testing methods is essential. The relative advantage of developmental toxicity testing using alternative methods is the less differentiated early embryonic developmental stages, making them comparable to actual developmental stages in animals [80]. Therefore, alternative methods provide highly accurate predictions, making animal tests based on rapid and precise alternative method screening results ideal for assessing developmental toxicity. The EBT, providing results in just four days with high accuracy, is the most suitable alternative method for early embryonic developmental toxicity (Table 9 and Figure 2).

### 3.2. Embryonic Developmental Toxicity Studies Using the EBT

After the second-phase validation updated the test method, three studies using the EBT for toxicity research have been reported. Jung et al. conducted a toxicity evaluation and examined the mechanisms of endocrine-disrupting chemicals (EDCs) using embryonic bodies (EBs) [76]. During the research process using EBs, the toxicity of 10 known EDCs (e.g., methyl and ethylparaben, triclosan, octylphenol, trichloroacetic acid, methoxychlor, bisphenol A, and diethylstilbestrol) was re-confirmed through the EBT. Furthermore, gene expression studies in the EBs verified the correlation between toxicity and ER stress. In the previous study, the EBT toxicity model demonstrated 90% accuracy, confirming its excellent predictive capabilities. Jeong et al. studied the cardiotoxicity of sodium arsenate (SA) using the embryoid body of mice [77]. In their study, the EBT was used to confirm the developmental toxicity level of SA, and the developmental toxicity was confirmed based on the score of the discriminant function (SDF) value of 2.59, which significantly exceeded the toxicity threshold (>−0.667). In 2023, Lee et al. conducted a pre-study on the effects of decamethylcyclopentasiloxane (D5) on the reproductive system of female rats using the EBT [78]. The EBT assessed the cell viability of mESC and 3T3-L1 and the reduction in the EB area, confirming the toxicity before proceeding with animal testing. In the EBT for D5 presented in this study, the SDF was 4.8, significantly exceeding the toxicity threshold (>−0.667), confirming the presence of developmental toxicity. As in previous studies where the EBT was used, the EBT can either replace animal tests for developmental toxicity or verify the presence of toxicity before conducting animal tests, minimizing unnecessary animal testing. In addition, the test can be used easily and expanded for various mechanistic studies.

The efficiency and accuracy of the embryoid body test (EBT) can be maximized through high-throughput screening (HTS). In 2024, Yixian et al. reported the development of an HTS method utilizing the EBT in a 384-well format [81]. This study tested the predictive accuracy and sensitivity of the developed model using the reference compounds outlined in the International Council for Harmonisation of Technical Requirements for Pharmaceuticals for Human Use (ICH) S5 (R3) guidelines, achieving an accuracy of 84.38%, sensitivity of 86.96%, and specificity of 77.78% across 32 compounds. Despite its relatively recent development and validation, the EBT is already being widely applied, demonstrating its excellence and indicating its potential for broader adoption in the future.

## 4. Limitations and Directions for Improvement in the EBT

### 4.1. Limitations—Alternative Developmental Stages and Species Differences

The applicability of the EBT is limited to specific periods because of the nature of alternative testing methods that simulate particular systems at specific times. In terms of utilizing the three-dimensional cellular structure, the EBT is similar to the organoid-based test. However, EBs represent structures from the early stages of embryonic development and are used to assess more general toxicity, such as the viability of embryos and the progression of development, due to the characteristics of these stages [45]. In contrast, organoids simulate the environment of a specific organ as closely as possible to investigate more specific and targeted toxicity [45,73]. The reliance on the undifferentiated state of embryoid bodies focuses on early differentiation processes but limits its application to modeling advanced stages of tissue development. Considering this undifferentiated state, the EBT is estimated to replace toxicity testing in the early development stages, approximately one to three weeks of pregnancy in humans [82]. Although this undifferentiated nature allows for differentiation through various factors, enabling adjustment and additional research, multiple directions exist depending on the purpose, requiring extensive validation to ensure accuracy.

The EBT, using mouse embryoid bodies, can replace mouse tests but does not directly represent human results. This limitation can lead to misunderstandings caused by species-specific toxicity differences, sometimes critically so. For example, the efficacy and toxicity of thalidomide arise from the degradation of neo-substrates through binding with CRBN, affecting the SALL4 protein and causing limb development issues [83]. On the other hand, this toxicity does not appear in mice or rats because of the different amino acid compositions in the CRBN gene (Table 10). Thalidomide was included among the test substances in the second validation test for the EBT, but it showed no toxicity, contrary to the results in humans. The literature also suggested that this difference in results may be due to differences in species-specific sensitivity [75]. These limitations are also an issue in animal testing, and the requirement for the test results from non-rodent animals, such as dogs or monkeys, in nonclinical studies is along the same lines. Nevertheless, it is not uncommon for this limitation to be challenging to overcome, which also serves as a basis for objections to animal testing.

To address interspecies differences, human-derived stem-cell-based tests have been developed in recent years [66,85,86,87,88]. However, they are still relatively complex and sensitive, with human embryonic stem cells (ESCs) facing ethical problems, and induced pluripotent stem cells (iPSCs) requiring additional costs and time [88]. As a result, tests like the EST or EBT using mouse-derived cells remain simpler and more practical, supported by extensive accumulated data and validation. Since the EST and EBT methods share a similar conceptual foundation, attempts to enhance the EST method using human stem cells could likewise be extended to the EBT method [87]. In the future, as the previously mentioned limitations are gradually overcome and the development and validation of testing methods using human stem cells are successfully achieved, this approach could establish itself as a more ultimate alternative testing method. It would address interspecies differences—challenges that even animal testing cannot overcome—while saving time and costs and providing greater accuracy.

### 4.2. Future Directions for the EBT

Like all alternative testing methods, the EBT has its advantages and disadvantages. One of the main advantages is that it uses embryoid bodies, which are closely associated with early embryonic developmental processes, allowing for rapid and easy testing. In addition, the use of these embryoid bodies enables the application of the test for studying mechanisms and even toxicity testing after differentiation. On the other hand, one of the key disadvantages is that, as a validated testing method, using the EBT to assess toxicity after differentiation is challenging.

To improve the method, if the test duration could be reduced by even one day while maintaining accuracy, the accessibility of the test could be maximized by leveraging its strengths. Furthermore, to overcome the limitation of only being able to assess toxicity in the undifferentiated state, establishing protocols for differentiation toxicity targeting major organs like the heart or brain/nervous system would enable the EBT to serve as the gold standard in embryonic developmental alternative toxicity testing [73].

## 5. Conclusions: Prospects of Alternative Testing Methods and the Role of the EBT

With the development of the pharmaceutical industry, the need for related toxicity testing is increasing continuously. On the other hand, as animal welfare and ethics gain importance, the regulation of animal experiments is tightening, and opposition to such experiments is growing. Consequently, the utilization and significance of alternative animal testing methods are expected to expand rapidly.

Recently, methods such as organ-on-a-chip, which aim to simulate the in vivo environment as closely as possible, have attracted increasing attention. In addition, efforts to predict results quickly and easily with minimal effort through in silico prediction models are ongoing. Nevertheless, more precise tests tend to incur costs as high as animal testing, and prediction models face limitations because of insufficient accumulated data and reduced reliability. Therefore, it is crucial to select the most appropriate testing method based on the specific purpose and focus to reduce animal testing, save time and costs, and obtain accurate toxicity data, while continuously improving each testing method to better align with its intended objectives. From this perspective, we aimed to develop and validate testing methods that are simple yet accurate, facilitating quick decision-making in the early stages of drug development.

**Figure 3:**
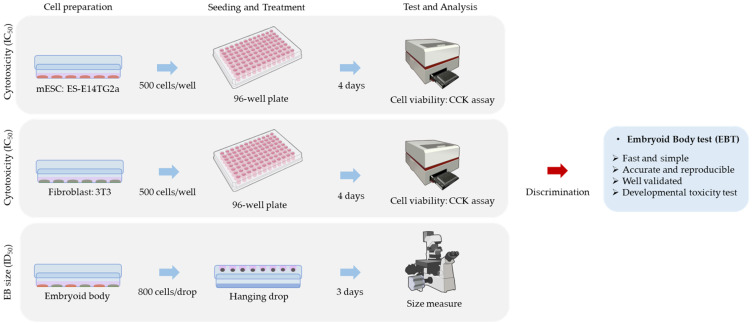
Test composition and features of the EBT.

The EBT is a relatively traditional in vitro test method that simplifies the process, minimizing time and cost while ensuring test accuracy (Figure 3). The test is a verified, up-to-date method with high reproducibility, making it easily usable by anyone at the screening stage or later for assessing early developmental toxicity. This test method clearly demonstrated the early embryonic developmental toxicity in mice, promoting reasonable research and development while adhering to animal welfare and ethics, and is expected to be used widely in the future.

## Figures and Tables

**Table 1:** Origin and development of major organs.

Organ	Germ Layer Origin	Germ Layer Time	Further Development
Heart	Mesoderm	~3 weeks	Primitive heart tube form (22 days), C-shape bending of heart tube (24 days), primordial division of the four chambers (28 days), and fetal heart completely formed (56 days) [4].
Brain	Ectoderm	~3 weeks	Neural tube forms (4 weeks), neurogenesis (4 weeks–postnatal), microglial entry (4–16 weeks), synaptogenesis (12 weeks–postnatal), apoptosis (18 weeks–postnatal), gliogenesis (22 weeks–postnatal), and myelination (32 weeks–postnatal) [5].
Lung	Endoderm and mesoderm	~4 weeks	Formation trachea and main bronchi (~7 weeks), branching airways (8–17 weeks), respiratory bronchiole, alveolar ducts, and primitive alveoli (18–27 weeks), alveoli and gas exchanging (24 weeks–birth), septation and multiplication of alveoli (36 weeks–2 years) [6].
Liver	Endoderm and mesoderm	~3 weeks	Liver diverticulum (23–26 days), liver bud (26–32 days), liver bud outgrowth (31–56 days), hepatocyte and cholangiocyte differentiation (56–210 days), gestation duration (266–280 days) [7].
Kidney	Mesoderm	~4 weeks	Formation of pronephros (4 weeks), mesonephros (4 weeks), metanephros (5 weeks), and glomeruli (9 weeks~). Nephrogenesis is complete by 36 weeks [8].
Gastrointestinal tract	Endoderm, mesoderm, and ectoderm	~3 weeks	Prime gut tube forms, gastrulation (3 weeks), close the cranial end of the digestive tube (4 weeks), peritoneal cavity develops (6 weeks) and rotates (10 weeks), obliteration of the vitelline duct (7 weeks), distal cloacal membrane opening and villus formation (9 weeks), distinctive longitudinal and circular muscle layers in the intestines (11 weeks), crypt development begins (12 weeks), muscularis mucosae develops (14 weeks), fetal intestinal absorption function develops (24 weeks) and reaches adult levels (32 weeks) [9].
Spleen	Mesoderm	~5 weeks	Appears as a bulge (5 weeks), columnar cell replacement and basement membrane present (6 weeks), apparent spleen formation (8 weeks), formation reticular framework, accumulation of lymphocytes and primitive white pulp (20~23 weeks), marginal zone appears in the reticular framework (26 weeks) [10].
Pancreas	Endoderm	~5 weeks	Formation of pancreatic buds (5 weeks), fusion of the buds (6 weeks), pancreatic duct joins duodenum (8 weeks), pancreatic tubules and clusters (9 weeks), lobule formation (14 weeks), acinar cells appear (12–15 weeks), and significant increase (20 weeks) [11].

**Table 3:** OECD test guidelines related to developmental toxicity.

No.	Title	Purpose	Animal Test
TG 414 [29]	Prenatal Developmental Toxicity Study	Assessing chemical effects on pregnant animals and fetuses.	Animal: Rodent (rat preferred) and pregnant non-rodent (rabbit preferred). Group: 1 control and 3 dose test groups (*n* > 20, pregnant and implantation sites at necropsy). Dosing: From implantation to one day before sacrifice, <1000 mg/kg/day.
TG 415 [30]	One-Generation Reproduction Toxicity	Assess test substances’ effect on male and female reproductive performance.	Animal: Rat or mouse. Group: 1 control and 3 dose test groups (*n* > 20, pregnant at/near term). Dosing: From growth to mating (male) or nursing period (female), <1000 mg/kg/day.
TG 416 [31]	Two-Generation Reproduction Toxicity	Assessing the effects of test substances on the performance of the reproductive system and on the growth and development of offspring.	Animal: Rat preferred. Group: 1 control and 3 dose test groups (*n* > 20, pregnant at/near parturition). Dosing: From growth to weening (parent and 1st generation), <1000 mg/kg/day.
TG 421 [32]	Reproduction and Developmental Toxicity Screening Test	Screen for potential reproductive and developmental toxicity of chemicals.	Animal: Rat. Group: At least 1 control and 3 dose test groups (*n* > 10, each sex). Dosing: >4 weeks for male, 63 days for female.
TG 422 [33]	Combined Repeated Dose Toxicity Study with the Reproduction and Developmental Toxicity Screening Test	Evaluate reproductive and developmental toxicity through repeated exposure.	Animal: Rat. Group: At least 1 control and 3 dose test groups (*n* > 10, each sex). Dosing: >4 weeks for male, 63 days for female.
TG 426 [34]	Developmental Neurotoxicity Study	Evaluate developmental neurotoxicity due to repeated exposure during pre/postnatal.	Animal: Rat preferred. Group: 1 control and 3 dose test groups (*n* > 20, litters). Dosing: From implantation (GD 6) to throughout lactation (PND21).
TG 443 [35]	Extended One-Generation Reproductive Toxicity Study	Assess reproductive, developmental, and systemic toxicity over one generation.	Animal: Rat preferred. Group: Cohort 1A/1B–20/sex/group; Cohort 2A/2B and 3–10/sex/group (1 control and 3 dose test groups). Dosing: From 2 weeks before mating to weening (parents), from prenatal to growth or weaning (1st generation).

**Table 5:** First-developed prediction model of the EBT (four classifications).

Discriminant Function: a $\;\log\;({\mathrm{IC}}_{50}\;3T3)-$ $\;b\;\log\;({\mathrm{IC}}_{50}\;\mathrm{mES})-$ $\;c\;\frac{IC50mES-ID50EB}{IC50mES}-d$					
Class	a	b	c	d	Judgment
Non	−0.907	0.992	0.022	3.746	The function of the class that produces the highest value
Weak	−0.242	1.886	0.021	4.299	
Moderate	0.541	4.062	0.042	9.856	
Strong	0.906	5.675	0.064	17.223	

**Table 6:** Changed prediction model of the EBT in pre-validation (two classifications).

Discriminant Function	Judgment
−0.4852526 × log (IC_50_ mES) − 0.5251880 × log (IC_50_ 3T3) + 0.1700371 × log (ID_50_ EB)	- Non-toxic ≤ −2.353739 - Toxic > −2.353739

**Table 7:** Final prediction model of the EBT (2 classification).

Discriminant Function	Judgment
−0.1139733 × log (IC_50_ mES) − 0.2303571 × log (IC_50_ 3T3) − 0.6726328 × log (ID_50_ EB) + 2.723601	- Non-toxic ≤ −0.667 - Toxic > −0.667

**Table 8:** Development and validation progress of the EBT method.

	Method Development	Pre-Validation	2nd Validation
Object	Develop a simple and accurate alternative developmental toxicity test.	Simplify and verify the test method	Update and verify the prediction model
Test outline (results)	Investigation of EB area reduction mechanism due to developmental toxicity (biomarker-decreased *Dnmt3b*, *Cbx5*, *Hdac2*, and increased *Cdkn2a*). Correlation investigation between EB area (%) and beating ratio of cardiac-differentiated EB (%) (r = 0.8652, *p* < 0.0001) Comparison of prediction accuracy between EST-PM and EBT-PM (accuracy EST: 86.9%, EBT: 90.5%) 21 compounds tested (6 toxic, 15 non-toxic)	Comparison of results by test period and shortening the test period (10→4 days) Intra-/Inter-laboratory reproducibility test (accuracy 84%/82%) Inter-laboratory transferability test (accuracy 92%) 26 compounds tested (9 toxic, 17 non-toxic)	Prediction model update and confirm using training set (accuracy 92%) Inter-laboratory reproducibility test (accuracy 87%) Predictive capacity test (accuracy 80%) 35 compounds tested (11 toxic, 24 non-toxic)
Significance	Successfully developed a more straightforward and more accurate test than the EST	Simplify testing to maximize accuracy and reproducibility	Improve accuracy with many test results

**Table 9:** Alternative developmental toxicity tests and their characteristics.

Method	Description	Advantages	Limitations	Applications
EST (embryonic stem cell test) [62,64]	The first widely accepted alternative test for developmental toxicity using mouse embryonic stem cells. Assessment of developmental toxicity based on stem cell and fibroblast cell toxicity, as well as cardiomyocyte differentiation.	Extensively studied over a long period: Improvement strategies have been devised (e.g., integration of FACS). Enables the evaluation of cardiomyocyte differentiation and functional effects.	Requires a differentiation induction process, which is relatively time-consuming and complex, reducing reproducibility. Improvement methods often require specialized techniques or equipment.	7–10 days
Hand1-Luc EST [67]	Luciferase reporter inserted into the Hand1 gene detects cardiomyocyte differentiation: Reducing the evaluation period for cardiomyocyte differentiation.	Shorter test duration and simplified procedure compared to the original EST: enhanced reproducibility and sensitivity. Enables rapid detection of cardiomyocyte differentiation toxicity.	Requires genetic modification. Hand1 is an early marker of cardiomyocyte differentiation, making it difficult to assess its functionality and teratogenicity.	5–6 days
EBT (embryoid body test) [73,74,75]	Assesses cytotoxicity in mouse stem cells and fibroblasts, as well as toxicity in their embryoid body formation.	Simple and shorter in duration, but with higher reproducibility and accuracy.	It may miss post-differentiation developmental toxicity.	4 days

**Table 10:** Thalidomide-binding sequences of CRBN and developmental toxicity by species [84].

Species	Binding Domain (Partial) in CRBN	Developmental Toxicity
Human	NLIGRPST**E**HSWFPGYAWT**V**AQC	Embryopathy, phocomelia
Rabbit	NLIGRPST**E**HSWFPGYAWT**V**AQC	Limb anomalies, embryo lethality
Chicken	NLSGRPST**E**HSWFPGYAWT**I**AQC	Limb defect
Mouse	NLIGRPST**V**HSWFPGYAWT**I**AQC	Non affected
Rat	NLIGRPST**V**HSWFPGYAWT**I**AQC	Non affected

Bold: The difference in amino acids between species.

## Data Availability

Not applicable.
